# Economic Impact of the Introduction and Establishment of *Drosophila suzukii* on Sweet Cherry Production in Switzerland

**DOI:** 10.3390/insects8010018

**Published:** 2017-02-08

**Authors:** Dominique Mazzi, Esther Bravin, Manuela Meraner, Robert Finger, Stefan Kuske

**Affiliations:** 1 Strategic Research Division Plant Protection, Agroscope, Schloss 1, 8820 Wädenswil, Switzerland; 2 Competence Division for Research Technology and Knowledge Exchange Plants and Plant Products, Agroscope, Schloss 1, 8820 Wädenswil, Switzerland; esther.bravin@agroscope.admin.ch (E.B.); stefan.kuske@agroscope.admin.ch (S.K.); 3 Agricultural Economics and Policy, Swiss Federal Institute of Technology (ETH), Sonneggstrasse 33, 8092 Zurich, Switzerland; mmeraner@ethz.ch (M.M.); rofinger@ethz.ch (R.F.)

**Keywords:** enclosure nets, harvest losses, insecticides, integrated pest management (IPM), invasive insect pests, mass trapping, sanitation, spotted wing drosophila, stone fruits, surveillance

## Abstract

First detected in Switzerland in 2011, the invasive *Drosophila suzukii*, spotted wing drosophila, has caused recurring costs for growers of berries and fruit. Recommended management approaches rely on a set of methods, tailored to suit crop requirements under the prevailing local conditions. Control of *D. suzukii* represents a substantial economic burden for growers, in terms of material, equipment, new infrastructure and extra labour. However, those growers who invest wisely to deliver unblemished produce are rewarded with high payoffs. We present insights from a growers’ survey conducted in 2015 and 2016 to gauge the impact of the introduction and establishment of *D. suzukii* on Swiss sweet cherry production. The surveyed growers (111 in 2015 and 298 in 2016) observed the recommended surveillance, sanitation and control measures. The use of insecticides (78% and 79% of respondents in 2015 and 2016, respectively) and the harvest of all fruits (93% and 59% of respondents in 2015 and 2016, respectively) were the most widespread methods used to reduce damage. Nearly one-third of the respondents set up enclosure nets. Our economic evaluation of different scenarios provides a quantitative indication of the potentially incurred costs. We argue for enhanced stakeholder involvement to raise the acceptance of integrated pest management practices, and to inform research and outreach by providing insights into the motivations and barriers to adoption.

## 1. Introduction

The newly invasive insect pest, *Drosophila suzukii* Matsumura (Diptera: Drosophilidae), spotted wing drosophila, is a devastating, highly polyphagous vinegar fly native to Southeast Asia. It was initially detected almost simultaneously in North America and Europe in 2008. Since then, it has spread rapidly to become a key pest damaging economically relevant soft and thin-skinned fruit crops in the major fruit production areas of the northern hemisphere [1,2,3] as well as in some South American countries [4]. Unlike other *Drosophila* species, which attack decaying or rotting fruit, *D. suzukii* females use a serrated ovipositor to lay eggs into intact, ripening fruit. The damage arises directly from oviposition wounds and internal larval feeding and indirectly from secondary pathogens, making infested fruit unmarketable [5,6].

Crop losses range from negligible to total depending, among other things, on crop, cultivar, farm location, intended channel of product distribution and market structures, making it difficult to put a price tag on the economic impact of *D. suzukii*. Nonetheless, first estimates of revenue losses in selected host crops provide an indication of the magnitude of the economic implications and altogether suggest that the benefits of management far outweigh the costs incurred if *D. suzukii* is not controlled [5]. A first evaluation of the economic impact of *D. suzukii* indicated that revenue losses due to *D. suzukii* in strawberry, blueberry, blackberry, raspberry and cherry production may exceed 500 million U.S. dollars (USD) in the three western states of the U.S.A.—California, Oregon and Washington—alone [7]. The study [7], however, did not account for growers’ adaptation responses. Goodhue, Bolda, Farnsworth, Williams and Zalom [5] examined potential revenue losses and control costs in Californian raspberry and strawberry production. Their analysis added to the existing literature by allowing for a price response, i.e., the increase in market prices due to reduced supply resulting from *D. suzukii* infestation. Benito et al. [8] estimated economic losses due to *D. suzukii* infestation in Brazil. Relying on spatial information on potential infestation of susceptible crops, they multiplied the potential yield losses by the expected revenue to generate aggregated figures on potential economic losses. They anticipated potential losses for peach and fig production amounting to about 30 million USD (21.4 and 7.8 million USD in peach and fig, respectively) [8]. In Europe, De Ros et al. [9] estimated the potential economic impact of *D. suzukii* on the production of strawberries, raspberries, blueberries, blackberries and cherries in the Italian region of Trentino. Their week-specific analysis accounted for the temporal aspects of production and infestation and put the total annual damage for the five crops examined in Trentino at over 3 million Euros (EUR). However, the analysis was limited to aggregated levels and neglected the costs of pest control. In a follow-up study, updated information was provided on infestation and damage recorded for the same region and crops over the period 2011–2013, and *D. suzukii* control costs were included [10]. Costs for materials, labour and infrastructure to implement surveillance, sanitation and pest control measures added up to total potential damage costs of one million EUR per year [10]. Ioriatti et al. [11] estimated that in 2010 berry growers incurred a 25%–35% reduction of production value, depending on crops (more for blueberries and raspberries). An additional estimated 500,000 EUR of losses were attributed to post-harvest sorting of fruit in the storage facilities and the shorter shelf life of contaminated fruit. Thus, the overall economic impact of *D. suzukii* damage in 2010 was estimated at 3–4 million EUR in the province of Trento alone [11].

The present study aims to assess rates of adoption of different *D. suzukii* management measures and to gauge the perceived benefits of adopting those measures. Using sweet cherry production in Switzerland as our basis, we present the insights obtained from a growers’ questionnaire survey, and complement them with an estimation of the costs sustained under different scenarios of *D. suzukii* infestation. Our discussion of the opportunities and constraints of adaptation and mitigation responses reflects the far-reaching impact of *D. suzukii* on an entire production sector. Our work can guide research and outreach activities by identifying determinants of pest management decision-making and provides an indication of the magnitude of the economic repercussions of a recent pest invasion.

## 2. Background

*D. suzukii* was first detected in Switzerland in 2011 [12]. While at first only sporadic local crop damage occurred, in 2014, *D. suzukii* imposed severe economic burdens on growers of berries (e.g., strawberries, raspberries, blueberries), stone fruit (e.g., cherries, plums, apricots) and grapes, thus raising much public concern and media attention. In the summer of 2015, a longer period with above-average temperatures and low precipitation, together with a strict implementation of preventive sanitation measures, provided some respite, even though trapping counts remained high throughout the season. In 2016, weather conditions were again favourable for the early build-up and continuous growth of *D. suzukii* populations. Adult trap captures were at least as high as in 2014 before and throughout the fruit growing season. However, the extent of crop damage did not reach the levels of 2014.

At present, no known control method meets the zero-tolerance policy for *D. suzukii* infestation demanded by the marketers. Hence, ongoing research efforts worldwide aim to develop sustainable practices to manage *D. suzukii* in commercial fruit crops by incorporating non-chemical management methods as part of an integrated strategy [13]. The management of *D. suzukii* requires a holistic approach, involving the many actors along the food production chain to ensure adequate post-harvest transport, storage and distribution, as well as access to the export markets. Furthermore, regulatory bodies must weigh the risk of economic crop losses against the risk of disrupting the equilibrium of horticultural crop ecosystems and the maintenance of well-established integrated pest management (IPM) programs.

In Switzerland, sweet cherries are grown either on dwarf and semi-dwarf rootstock trees or on high-stem trees and are produced as table cherries for fresh consumption, for confection and bakery products, fruit juices and syrup production, canning and distillation. Annual sweet cherry production has fluctuated substantially in recent years, ranging from a peak of 19,000 tons (in 2000) to a low of 5500 tons (in 2013) (Swiss Farmers’ Union, www.sbv-usp.ch). The annual *per capita* sweet cherry consumption in Switzerland amounts to around 1 kg (Swiss Farmers’ Union, www.sbv-usp.ch). The consumption of sweet cherries has steadily increased within the last fifteen years and the domestic production covers about one third of the demand [14]. Table 1 gives the year-to-year variation in the relative proportion of sweet cherry production destined for the fresh market, for processing and for distillation. The proportion of cherries for distilling includes those produced for other purposes, but which fail to meet the quality standards. In 2015, the indicative retail prices for one kilogram of table cherries were 6.50 Swiss Francs (CHF) (class 1, 21 mm), 8.50 CHF (extra class, 24 mm) and 11 CHF (premium class, 28 mm); cherries for processing sold at a recommended price of 4 CHF/kg and cherries for distillation at 1.50 CHF/kg [14]. Due to these differences in output price, the expected returns from crop protection measures as well as the expected adoption of such measures differ across the intended target market. Table cherries have a yearly production value of around 30 million CHF (Swiss Federal Office for Agriculture fruit crops statistics).

Table cherries are grown in orchards. The trees are typically protected from severe weather with plastic shields and hail nets. Many orchards have additional protective side nets against birds. Trees are pruned regularly and watered with micro sprinklers or drip irrigation. The control of *D. suzukii* in these commercial sweet cherry orchards relies increasingly on the use of enclosure nets covering fruit bearing trees. Recommended mesh sizes vary [15], but mesh sizes around 1–1.3 mm limit the risk of *D. suzukii* damage by reducing and delaying infestation without negatively altering the microclimate under the net or increasing exposure to strong wind [16,17]. Baited traps placed inside enclosure nets and insecticides may provide extra control. Infestation of fruits on unprotected orchard trees and high-stem trees can only be reduced if sanitation measures (e.g., the timely removal of fallen and overripe fruit, the proper disposal of contaminated fruit, the shortening of harvest intervals and the clearing of ground covering vegetation) are implemented scrupulously and, when unavoidable, by the use of authorized insecticides (spynosins, neonicotinoids and pyrethrins).

This study is part of an ongoing multi-stakeholder program directed at mitigating the economic impact of *D. suzukii* on Swiss horticulture. Following the serious economic damage from *D. suzukii* sustained in 2014 in many crops (e.g., raspberries, blackberries, cherries, apricots, plums, grapes), the Swiss parliament allocated extraordinary funding to a task-force (www.drosophilasuzukii.agroscope.ch) charged with bundling the efforts of all concerned actors in the quest for solutions to minimize the damage inflicted by *D. suzukii* on the production and marketing of berries, stone fruit and grapes. The task-force pursues both application-oriented (“extension”) research activities addressing the immediate demands of growers as well as fundamental research activities on pest ecology designed to support the development of sustainable management solutions.

## 3. Materials and Methods

We surveyed Swiss sweet cherry growers using an online questionnaire. The survey was developed in cooperation with extension specialists and advisors and implemented using the online tool Q-Set (www.q-set.ch). Following ten pre-tests, which generated important feedback to improve the survey, the regional advisory services distributed the survey to all known Swiss sweet cherry growers in early July (i.e., after the closing of the harvest season) via personalized e-mails with an explanatory cover letter. A follow-up reminder was mailed to all recipients 5–6 weeks after the initial mailing. The survey closed at the beginning of September. The link to the online survey was provided in all mailings and a printable PDF version was attached. The survey was further advertised at growers’ meetings and events, as well as in the printed and online channels through which plant protection recommendations are disseminated during the fruit growing season, including a bi-monthly newsletter issued by the federal research station Agroscope. There was a draw for four 50 CHF gift vouchers to encourage participation. The survey was conducted in 2015 and in 2016.

The survey included an introductory section providing details about the aims of the project, an assurance of confidentiality and a note about eligibility. The questions addressed fell into three categories. Firstly, participants were asked general questions about the characteristics of their business, such as geographical location, cultivated area for each cultivar and tree size. Secondly, they were requested to provide information about *D. suzukii* fruit infestation in terms of the number of infested cherries in a randomly picked sample of fifty intact ripe cherries and of the number of deliveries rejected by traders, if any. Thirdly, they were asked to select the implemented *D. suzukii* management measures from a list of surveillance, sanitation and control methods and to rate their satisfaction with the effectiveness of the measures. At the end of the questionnaire, participants could provide additional information.

Following the evaluation of the responses collected in 2015, the questionnaire for the 2016 survey was modified slightly and refined to better match the objectives and also to clear up some misunderstandings that had become apparent. In particular, while in 2015 growers were asked about the implemented set of management measures on their entire farm, in 2016, they were asked about the set of measures implemented for each sweet cherry cultivar (i.e., for each plot), to account for adjustments to cultivars (i.e., to their time of ripeness and thus, ultimately, their susceptibility). Consequently, in 2015, growers were asked how satisfied they were with the effectiveness of single measures on the entire farm, while, in 2016, they were asked to grade their satisfaction with the effectiveness of different sets of measures applied to different plots. Additionally, while in 2015 we asked about the growers’ satisfaction with the effectiveness of the measures implemented using a yes/no answer (with the answer choices “yes”, “partly”, “no” and “do not know”), in 2016, we asked growers to rate the measures on a five-point scale (from 1: “very unsatisfied” to 5: “very satisfied”). The 2016 survey is available in the original German as Supplementary Material. We use descriptive statistics to summarize key features of the information collected from the growers’ survey. Note that sample sizes vary due to missing values because not all respondents answered all the questions. We used quantitative information obtained from growers, advisors, growers’ and trading associations, as well as from the Federal Office for Agriculture to estimate the sustained costs under different scenarios of fruit infestation. As of 30 November 2016, the exchange rate for one U.S. dollar (USD) is equal to 0.94 Euros (EUR) and 1.01 Swiss Francs (CHF).

## 4. Outcomes from the Growers’ Survey

An overview of the surveyed characteristics of the responding growers (*N* = 111 and *N* = 298 in 2015 and 2016, respectively) and their managed sweet cherry crops are provided in Table 2.

The vast majority of respondents carried out surveillance, sanitation and control measures to manage the risk of *D. suzukii* infesting their crops (Table 3). Most respondents (82% in 2015 and 88% in 2016) practiced more than one management measure during one cropping season (Table 4). The prevailing strategy was the combination of insecticide use and harvest of all fruit in both years. Growers applied up to three insecticide treatments in a combined strategy aimed to control both the European cherry fruit fly *Rhagoletis cerasi* L. (Diptera: Tephritidae) and *D. suzukii*. In 2016, for example, 37% of the 205 respondents carried out three insecticide treatments, 23% two treatments and 19% one treatment, whereby one treatment with the active ingredient spinosad targeted *D. suzukii*, while additional treatments with the active ingredients acetamiprid and thiacloprid may have been primarily directed against *R. cerasi*.

Many respondents suffered fruit damage from *D. suzukii*, despite the implemented management measures. In 2015, 56% of the respondents who had performed the requested check of a sample of fifty cherries (*N* = 48) reported at least some *D. suzukii* fruit infestation and 12% had at least one fruit delivery rejected by traders because of *D. suzukii* infestation (*N* = 94 growers provided an answer). In 2016, 88% of respondents reported at least some *D. suzukii* fruit infestation (*N* = 269 valid answers provided) and 23% had at least one fruit delivery rejected by traders because of *D. suzukii* infestation (*N* = 279 valid answers provided). The more valuable table cherries, grown on dwarf and semi-dwarf rootstock trees, and often protected with enclosure nets, were less damaged than cherries for processing and distilling grown on high-stem trees. For example, in 2016, 45% of the dwarf and semi-dwarf rootstock tree plots were infested, as opposed to 82% of the high-stem tree plots. Early ripening sweet cherry cultivars are harvested before a significant build-up of *D. suzukii* populations can occur and hence typically suffer less damage than later ripening cultivars. For example, in 2016, fewer plots of the early cultivars Bigarreau Burlat (24%, *N* = 78), Grace Star (28%, *N* = 23) and Merchant (30%, *N* = 128) were damaged than plots of the later cultivars Schauenberger (92%, *N* = 72), Star (75%, *N* = 84) and Sweetheart (72%, *N* = 25).

When asked about their satisfaction with the effectiveness of single control measures (in 2015), growers gave enclosure nets the highest rating. Out of the respondents who had used enclosure nets (*N* = 51), 65% declared themselves satisfied with their effectiveness. Out of the surveyed growers who had used insecticides (*N* = 86), 56% indicated that they were satisfied with their effectiveness. Mass trapping was rated lowest, with only 15% of users (*N* = 26) declaring themselves satisfied with its effectiveness. Indeed, the proportion of surveyed growers who implemented mass trapping dropped from 58% in 2015 to 20% in 2016. In 2016, the survey asked growers to rate their satisfaction with the effectiveness of sets of management measures. Thirty-seven respondents were very satisfied with the implemented strategy in all cultivar plots (i.e., rated all cultivar plots with the highest satisfaction score on a five-point scale, where 1 = very unsatisfied and 5 = very satisfied). Twenty-seven of them used insecticides, none of them used enclosure nets and two of them included mass trapping. Twenty-nine respondents were very unsatisfied with the implemented strategy in all cultivar plots (i.e., rated all cultivar plots with the lowest satisfaction score on the five-point scale). Twenty-two of them used insecticides, eight enclosure nets and seven mass trapping. In 2016, 156 growers answered an open-ended question asking which strategy they will use in future given they were not satisfied with the strategy implemented at present. In addition, 34% of the responses can be categorised as “intensify the use of insecticides”, 21% as “clear high-stem trees”, 17% as “intensify the use of enclosure nets”, 13% as “intensify the use of sanitation measures”, 10% as “clear dwarf and semi-dwarf rootstock trees” and 3% as “intensify the use of control measures”.

## 5. Estimation of the Sustained Costs Depending on the Extent of Fruit Infestation

Visual inspection of one 1-ha sweet cherry plot for *D. suzukii* infestation requires about ten labour hours (Markus Hunkeler, head of the plant protection advisory service of Canton Lucerne, cited in Bravin et al. [18]). Based on figures from the Swiss Fruit Association (www.swissfruit.ch), we assume growers’ hourly labour (opportunity) costs of 35 CHF and thus the total expenditure for checking fruit for *D. suzukii* infestation amounts to 350 CHF/ha. Setting up surveillance traps costs about 8 CHF/ha (Table 5). These costs are calculated according to Hunkeler in Bravin, Gremminger and Peterhans [18] based on a price of 0.85 CHF/trap, a required trap density of four traps per ha, and 0.2 h/ha of labour to hang the traps, again using labour costs of 35 CHF/h.

The costs for control measures include materials, labour and machines to apply insecticides, deploy mass trapping devices and mount and maintain enclosure nets. We used the business management simulation program Arbokost [19] to calculate the costs of the insecticide treatment. The model is used by growers and extension services to calculate full cost accounts, cash flow and returns of fruit production in Switzerland. The model, including all specifications, can be downloaded at https://www.agroscope.admin.ch/agroscope/de/home/themen/pflanzenbau/obstbau/oekonomie-obstbau/arbokost.html). Arbokost predicts costs of 312 CHF/ha for the insecticide treatment (Table 5). Mass trapping costs about 770 CHF/ha [18], assuming that growers use 330 traps/ha and need 12 h/ha to hang the traps. The investment for enclosure nets with a guaranteed life of ten years is 2000 CHF (i.e., 200 CHF/year), provided that rain covers and bird nets are already in place [20]. We assumed that two employees with an hourly salary of 21 CHF [14] need ten person hours to mount the nets. Hence, the total yearly costs for the enclosure of an orchard of dwarf or semi-dwarf rootstock trees already protected with rain cover and bird netting are 410 CHF/ha (Table 5). Based on the values given in Table 5, a minimum effort strategy consisting of visual fruit checks and the use of one insecticide treatment generates costs of 662 CHF/ha, while a maximum effort strategy consisting of visual fruit checks, surveillance trapping, one insecticide treatment, mass trapping and enclosure nets generates costs of 1857 CHF/ha (Table 5).

Costs for sanitation measures are not included in our calculations. As some measures would be implemented anyway, it is hard to assess the extra costs due specifically to *D. suzukii*. Growers did complain about an increase in workload, but its quantification is also unreliable because unpaid volunteers and family workers often participate in these tasks. Therefore, our calculations underestimate the real management costs to some extent.

Production costs rise further in step with the extent of *D. suzukii* infestation of fruit ready for harvesting. Once a plot is infested, harvesting costs rise as shown in Table 6 because additional effort is needed to identify and remove infested fruit. When fruit infestation is detected, it must be decided quickly whether the crop is worth harvesting or not. Growers typically harvest slightly infested sweet cherry crops (up to about 20% of the fruit attacked, often less). However, the careful inspection of fruit and sorting out of damaged fruit at harvest are time-consuming and the harvesting performance drops accordingly. In our calculations, we assumed a 50% drop in harvesting performance as compared to the performance achievable in the absence of infestation (i.e., a maximum of 6 instead of 12 kg/person hour). Growers typically forfeit harvesting heavily infested sweet cherry crops (over 20% of the fruit attacked) because the inspection and selection of fruits become too time-consuming and thus too expensive. In addition, the effort involved cannot be justified, since there is still a risk that infestation may be overlooked and the produce rejected at delivery. Growers are prepared to forfeit the yield of the plot and declare a 100% loss. This decision depends on the costs and availability of labour, the infestation rate of other plots, the sweet cherry surface of the farm, the production mix of the farm, the expected price for sweet cherries and the long-term consequences of not delivering to the market, which can compromise a privileged relationship with the traders. In this case, fruit quality no longer matters and fruit is gathered up to prevent it acting as a reservoir for later infestation. Thus, harvest performance can be increased to 40 kg/h to cut harvest costs beyond those incurred for non-infested plots, where harvest performance only reaches 12 kg/h (see Table 6). The increase in harvest performance lowers the production costs relative to a scenario of low infestation. However, the yield must eventually be disposed of and the growers obtain no returns to cover the costs. The loss caused by *D. suzukii* will be equal to the production costs. In this case, *D. suzukii* is estimated to generate a yearly loss for growers of 44,000 CHF/ha, depending on the implemented pest management strategies. If growers overlook infestation, the contaminated produce may be rejected at delivery. The tolerance rate is virtually 0%. When fruit is found to be infested with *D. suzukii*, the entire lot delivered for sale is destroyed. This is, in fact, the worst-case scenario: growers bear all the production costs, including harvesting costs for high-quality fruit, but obtain no revenues and must pay the extra costs for transportation, grading and disposal. In this case, the loss incurred due to *D. suzukii* will reach 71,000 CHF/ha. Table 7 shows the model-generated costs caused by *D. suzukii* under four scenarios of increasingly severe fruit infestation.

## 6. Discussion and Outlook

The rapid spread of *D. suzukii* across many fruit production regions has disrupted well-established, reliable, integrated pest management systems. This means that the fruit industries affected must adjust their production methods in order to meet this new challenge. In a time when great efforts are being made to develop effective and economically viable control programmes, our case study on pest management decisions of Swiss sweet cherry growers provides an overview of the status quo, and an outlook on the forthcoming development of *D. suzukii* management in an exemplary fruit production sector.

The formidable threat posed by *D. suzukii* has increased sweet cherry production costs considerably and indeed far overshadows the management issues regarding hitherto prevailing pests and pathogens. Our survey of Swiss sweet cherry growers confirms that the ubiquitous distribution of *D. suzukii* and its capacity to rapidly build up and sustain large populations put all commercial sweet cherry production operations at risk and force growers to budget greater expenditures for pest management. All of the management measures are time-, labour- and cost-intensive and, on their own, insufficient to prevent damage. Furthermore, these measures must be implemented early in the cropping season, regardless of their demonstrated need or benefit. Therefore, the mere presence of *D. suzukii* in a production area justifies expenditure on management costs and costs arise even in years in which other factors (mainly weather conditions) eventually delay or mitigate the effective pest damage. It is essential that management practices should be designed to cover large adjacent areas of cropland and also to target the pest outside of the fruit-growing period. Furthermore, particular consideration must be given to scattered unmanaged fruit trees, abandoned orchards, hedges, private gardens and other habitats providing alternative additional hosts.

Many of the sweet cherry growers who responded used baited traps to monitor the occurrence of adult *D. suzukii* on their farm and inspected the fruit for *D. suzukii* infestation. While adult captures are only a poor indicator of actual crop infestation risk [22] and thus do not replace the more laborious fruit checks, traps provide early warning of fly activity and insight into the temporal variation of *D. suzukii* population dynamics. Ongoing research efforts towards developing modelling tools that describe and forecast *D. suzukii* populations will improve future management practices by predicting pest pressure independent of trap captures and samples of infested fruit [1,23,24]. In the meantime, however, the surveillance measures currently in use remain the key to a more appropriate scheduling of control measures, in particular to more rational insecticide use strategies.

Growers have become aware of the importance of field sanitation as an essential element of any *D. suzukii* management program [6,13,25]. The adoption of sanitation measures is widespread in our sample of growers and includes timely, or even early, harvesting of all fruit and the removal and destruction of overripe, infested or culled fruit. Sanitation removes reservoirs for the build-up of on-farm populations, slows down *D. suzukii* population growth and spread and thus reduces pest pressure on later susceptible crops.

The use of insecticides (with the active ingredients spinosad, acetamiprid and thiacloprid) was the most common control measure adopted by the surveyed sweet cherry growers, in spite of the fact that it is only recommended as a last resort to avoid economic losses when all other control measures fail. The proportion of growers applying insecticides remained constant over the two survey years, suggesting that the decision to resort to insecticides was largely independent of the real crop infestation risk. Insecticides with some effectiveness against *D. suzukii* include organophosphates, pyrethrins and spinosyns; at present, an effective insecticide class for suppression of *D. suzukii* is perceived to be spinosyns (spinosad and spinetoram) while the neonicotinoid insecticides tend to be less effective against adults [26,27,28,29]. The narrow range of options, particularly in organic production, requires that insecticide treatments are carefully optimized in order to prolong their effectiveness and counter the development of resistance [29,30]. Frequent, prophylactic, and sometimes superfluous insecticide applications add to the growers’ private costs and also have negative external effects due to detrimental impacts on the environment and human health [31]. Furthermore, consumer trust may be undermined in a time of growing awareness for health and environmental issues in the food chain [32,33].

Mass trapping relies on the deployment of a large number of baited traps with the aim to physically remove flies from local populations and thus reduce pest pressure. The traps used may be the same or similar to those used for surveillance. Custom-made traps usually consist of plastic containers of varying shapes and colours with a number of small entry holes and baits are made of a mixture of apple cider vinegar, red wine and sugar [34,35,36,37]. So far, no affordable attractant can compete with ripe sweet cherries. Hence, mass trapping is expected to be most effective in late winter and early spring, before the fruit ripens, as well as after harvest, i.e., whenever cues from suitable fruit are absent. Placed early under enclosure nets, baited traps may remove flies already present in the crop, or inadvertently allowed to enter the enclosure. Traps may also help to “clean up” harvested plots. As a beneficial side effect, trapping survivors of insecticide treatments should help to slow down the development of insecticide resistance.

The exclusion approach using fine-meshed enclosure nets aims to prevent *D. suzukii* access to crops. A growing body of evidence attests to its effectiveness for reducing and/or delaying fruit infestation, with little or no effect on fruit quality, yield or damage from other pests and pathogens, particularly in berry crops [38,39] and also in sweet cherry crops [16,17]. The main barriers to the adoption of enclosure nets may be the perceived high initial investment costs and the lowered efficiency and worker comfort in commercial operations that require frequent access to crops for harvesting and maintenance. The risk of having to forfeit entire crops, or no longer being able to sell fruit that meets consumer expectations may well offset the significant costs of the fabric, installation, maintenance and repairs, as well as the hazards posed by pests and diseases potentially favoured by an altered microclimate under the net (e.g., spider mites, woolly apple aphid and powdery mildew). The cost of covering a plot of table cherries with insect netting depends on the support system used. In general, however, given a manufacturer’s guaranteed life of the net of ten years, enclosure nets are most probably a rewarding measure when the amortized cost of the investment is compared with the annual costs associated with *D. suzukii* infestation [17]. Swiss sweet cherry growers have rapidly recognized the appeal of returns gained from producing higher quality fruit, greater yields, with lower production and delivery risks and reduced need for insecticides. Within the last 4–5 years, about one in every three surveyed growers supplemented weather protection infrastructures by mounting additional enclosure nets to afford the extra protection from *D. suzukii*. The benefits to consumers in terms of fruit quality and yield will also need to be the cornerstones of a communication strategy addressing the visual impact of enclosure nets. As they become more widespread, efforts must be made to ensure that a perceived impact upon the local visual amenity and tourism assets does not compromise community acceptance.

We have presented a first descriptive analysis of growers’ attitudes towards the adaptation responses induced by the sudden and unexpected threat of a newly invasive, destructive insect pest of fruit crops in a representative affected production sector. We used additional stakeholder-derived information to provide a snapshot of the current state and future trends in the implementation of *D. suzukii* management and an estimation of the economic burden that has to be borne to secure the viability and sustainability of a locally significant branch of horticultural production. Our holistic approach to tackling the costs of *D. suzukii* infestation is, to our knowledge, unique and makes direct comparisons with earlier research a challenge for future studies. In the scope of an ongoing project (DROSOPHRISK), our future research in this area will address the determinants of the heterogeneous choices of risk management strategies, the growers’ subjective risk perception and risk preferences [40]. Characteristics of the growers and the business they manage are likely to influence the specific choice of certain *D. suzukii* management methods from a set of available management methods and ultimately determine farm-wide pest management strategies. Growers have a number of options for managing risk and different risk management tools are often utilized simultaneously. Hence, a holistic analysis of risk management decisions must include other coping strategies, such as the degree of specialization of the business, insurances and off-farm employment [41]. The continued survey and mapping of growers of sweet cherries and of other crops vulnerable to *D. suzukii* infestation will serve as an exemplary case study to uncover the factors underlying risk management decisions and track the effectiveness of actions taken to manage risk.

In addition to growers, many other actors in the agricultural sector incur costs from *D. suzukii* invasion, such as for the extra allocation of resources to research and its dissemination, information, education, consultancy and regulation, as well as for post-harvest handling, storage, distribution and marketing. Therefore, coordinating and reconciling different interests and expectations is vital for the success of an interdisciplinary, multi-stakeholder project explicitly directed at rapid problem-solving. We are convinced that the careful consideration of stakeholders’ views and the continuing evaluation of their knowledge, attitude and practice can enhance pest management along the entire food value chain and inform research priorities, outreach activities and policy-making.

## Figures and Tables

**Table 1:** Proportion (in %) of the Swiss sweet cherry production assigned to table cherries for fresh consumption, cherries for processing and cherries for distillation in 2012–2015 (Source: Swiss Farmers’ Union, www.sbv-usp.ch).

Product	Year			
	2012	2013	2014	2015
Table cherries	65	40	21	54
Processing cherries	5	15	17	8
Distillation cherries	30	45	62	38

**Table 2:** Summary statistics of selected characteristics of responding growers and their sweet cherry crops in two consecutive years.

Characteristics	2015	2016
Number of respondents (proportion of sweet cherry growers (%))	111 (10)	298 (28)
Average cultivated surface per respondent (ha)	0.8	1.0
Proportion of plots planted with dwarf and semi-dwarf rootstock trees (%)	70	53
Proportion of respondents from the German/French-speaking regions (%)	97/3	95/5

**Table 3:** Adoption of single pest management measures against *Drosophila suzukii* in a sample of Swiss sweet cherry growers in two consecutive years (*N* = 111 and *N* = 298 in 2015 and 2016, respectively). Measures are listed in descending order of frequency of use.

Pest Management Measures	Practiced Single Measures (% of Respondents)	
	2015	2016
**Surveillance measures**		
Visual fruit checks	83	63
Monitoring with baited traps	61	40
**Sanitation measures**		
Harvest of all fruit	93	59
Post-harvest removal of fallen fruit	43	46
Harvest ahead of time	43	35
**Control measures**		
Insecticide use	78	79
Mass trapping	58	20
Enclosure nets	32	31

**Table 4:** Adoption of combinations of pest management measures against *Drosophila suzukii* in a sample of Swiss sweet cherry growers in two consecutive years (*N* = 111 and *N* = 298 in 2015 and 2016, respectively). Combinations of measures are listed in descending order of frequency of use.

Combinations of Pest Management Measures	Practiced Combinations of Measures (% of Respondents)	
	2015	2016
Insecticide use and harvest of all fruit	32	28
Insecticide use, enclosure nets and harvest of all fruit	31	21
Insecticide use and enclosure nets	7	5
Enclosure nets and harvest of all fruit	7	3

**Table 5:** Costs of pest management measures against *Drosophila suzukii* in sweet cherry production in Switzerland.

Pest Management Measures	Materials (CHF/ha)	Labour ^6^ (CHF/ha)	Machines (CHF/ha)	Total (CHF/ha)
**Surveillance measures**				
Visual fruit checks ^1^	-	350	-	350
Monitoring with baited traps ^1,2^	4	7	-	11
**Control measures**				
Insecticide use ^3^	190	35	87	312
Mass trapping ^1,4^	522	252	-	774
Enclosure nets ^5^	200	210	-	410
**All measures**	916	854	87	1857

^1^ Markus Hunkeler, head of the plant protection advisory service of Canton Lucerne in Bravin, Gremminger and Peterhans [18]; ^2^ M. Schmid, head of the experimental fruit farm of Agroscope in Wädenswil, Switzerland and T. Schwizer, head of the stone fruit centre Breitenhof, Wintersingen, Switzerland; ^3^ Assuming one treatment with a product based on the active ingredient spinosad; ^4^ Ready-to-use traps consisting of a small transparent cup with holes pierced in their aluminium lid, filled with a liquid bait of wine, sugar and wine and fruit vinegar are commercially available in Switzerland for a price of 0.85 CHF per piece (www.becherfalle.ch); ^5^ [18]; ^6^ Labour costs are put at 35 CHF/ha (insecticide application) and 21 CHF/ha for all other tasks (Swiss Fruit Association, www.swissfruit.ch). Machine costs are put at 38 CHF/h (tractor) and 49 CHF/h (sprayer) [21].

**Table 6:** Harvesting costs depending on the extent of fruit infestation by *Drosophila suzukii* in sweet cherry production in Switzerland.

Proportion of Infested Fruit (%)	Performance ^1^ (kg/h)	Time Required ^2^ (h/ha)	Labour Costs ^3^ (CHF/h)	Total Costs (CHF/ha)
0%	12	1000	22	22,000
1% to 20%	6	2000	22	44,000
more than 20%	40	300	22	6600

^1^ and ^2^ Estimated on the basis of standard values set by an advisory panel made up of representatives of selected Swiss cantonal authorities and implemented in the model Arbokost; ^3^ Calculated on the basis of standard costs set by an advisory panel made up of representatives of selected Swiss cantonal authorities and implemented in the model Arbokost; Calculated amounts were rounded up to the next thousand.

**Table 7:** Estimation of the costs sustained due to the occurrence of *Drosophila suzukii* in sweet cherry production areas of Switzerland under four scenarios of increasing fruit infestation.

Proportion of Infested Fruit (%)	Pest Management	Additional Harvest Costs	Harvest Disposal	Delivery Disposal	Costs (CHF/ha)
0%	2000				2000 ^1^
≤20% at harvest	2000	22,000			24,000 ^2^
>20% at harvest	2000		42,000		44,000 ^3^
>0% at delivery	2000			69,000	71,000 ^4^

^1^ Sum of the costs for the surveillance measures, an insecticide treatment, mass trapping and enclosure nets, as given in Table 5. Calculated amounts were rounded up to the next thousand; ^2^ Difference between harvesting costs without infestation (0%) and with infestation (5%–20%), plus the costs of surveillance and control measures as in Table 5; ^3^ Arbokost calculation of production costs, assuming a harvest performance of 40 kg/h and standard values for yield, direct and structure costs; ^4^ Arbokost calculation of production costs, assuming a harvest performance of 12 kg/h and standard values for yield, direct and structure costs and additional grading costs of 6000 CHF/ha.

## Supplementary Materials

The supplementary materials are available online at http://www.mdpi.com/2075-4450/8/1/18/s1.
