# Supplementary material for: Economic Impact of the Introduction and Establishment of Drosophila suzukii on Sweet Cherry Production in Switzerland
Source: Insects. 2017 Feb 8;8(1):18. doi: 10.3390/insects8010018 (PMC5371946; doi:10.3390/insects8010018)
Supplement: Supplementary file 1 [file insects-08-00018-s001.pdf]

Bewertung der Kirschenschäden durch die Kirschessigfliege (KEF)

Sehr geehrte Obstproduzenten,
2015 führte Agroscope gemeinsam mit den kantonalen Fachstellen eine Umfrage bei Steinobstproduzenten durch. Daraus sind wichtige Erkenntnisse für Forschung, Beratung und Praxis entstanden. 2016 wird diese Umfrage wiederholt. Die Ziele der Umfrage sind den Ausfall zu bewerten und die Massnahmen der Praxis zu beurteilen.
Im Folgenden erwarten Sie Fragen zu den Bereichen:
- Kirschsortenbestand
- Bewertung der Schäden
- Massnahmen gegen den Befall
- Informationen zum Betrieb
- Informationen zum Betriebsleiter

Das Ausfüllen des Fragebogens dauert ca 20 Minuten.
Ihre Angaben werden vertraulich behandelt.

Wir bedanken uns schon jetzt für Ihre Zusammenarbeit!

Für Fragen kontaktieren Sie bitte:

Esther Bravin - Agroscope
esther.bravin@agroscope.admin.ch
Tel: 058 460 62 44

***

Die Umfrage endet am 18.09.2016

***

Das Zwischenspeichern des Fragebogens ist möglich. Es wird ab Seite zwei des Fragebogens ein Link am oberen Rand des Fragebogens eingeblendet. Alle Informationen bis zur vorherigen Seite werden gespeichert.

1 Welche Kirschensorten bzw. Kirschentyp haben Sie auf Ihren Betrieb? (Pflichtfrage)

(Mehrfachnennungen sind möglich)

- Bigarreau Burlat ☐
- Carmen ☐
- Christiana ☐
- Gardel (Coralise®) ☐
- Giorgia ☐
- Grace Star ☐
- Kordia ☐
- Merchant ☐
- Oktavia ☐
- Regina ☐
- Schauenburger ☐
- Star ☐
- Summit ☐
- Sweethart (= Sumtare) ☐
- Techlovan ☐
- Vanda ☐
- Verarbeitung- und Schüttelkirschen ☐
- Brennkirschen ☐
- Andere ____________ ☐

2 Bitte geben Sie Ihre Fläche pro Sorte (in Aren) an : (Pflichtfrage)

Fläche pro Sorte:

- Bigarreau Burlat ____________
- Carmen ____________
- Christiana

Gardel (Coralise®)	
Giorgia	
Grace Star	
Kordia	
Merchant	
Oktavia	
Regina	
Schauenburger	
Star	
Summit	
Sweethart (= Sumtare)	
Techlovan	
Vanda	
Verarbeitung- und Schüttelkirschen	
Brennkirschen	
Andere	

3 Bitte geben Sie die Zuchtform der Anlage pro Sorte an:

	Niederstammanlage	Feldobstbau/Hochstamm
Bigarreau Burlat	☐	☐
Carmen	☐	☐
Christiana	☐	☐
Gardel (Coralise®)	☐	☐
Giorgia	☐	☐
Grace Star	☐	☐
Kordia	☐	☐
Merchant	☐	☐
Oktavia	☐	☐
Regina	☐	☐
Schauenburger	☐	☐
Star	☐	☐
Summit	☐	☐
Sweethart (= Sumtare)	☐	☐
Techlovan	☐	☐
Vanda	☐	☐
Verarbeitung- und Schüttelkirschen	☐	☐
Brennkirschen	☐	☐
Andere	☐	☐

4 Welche Strategien haben Sie pro Sorte gegen die Kirschessigfliege eingesetzt? (Pflichtfrage)

	Monitoring mit Fallen	Insektizid	Hygiene-massnahmen bei der Ernte	Massenfang mit Becherfallen	Keine von diesen Strategien eingesetzt
Bigarreau Burlat	☐	☐	☐	☐	☐
Carmen	☐	☐	☐	☐	☐
Christiana	☐	☐	☐	☐	☐

Gardel (Coralise®)	☐	☐	☐	☐	☐
Giorgia	☐	☐	☐	☐	☐
Grace Star	☐	☐	☐	☐	☐
Kordia	☐	☐	☐	☐	☐
Merchant	☐	☐	☐	☐	☐
Oktavia	☐	☐	☐	☐	☐
Regina	☐	☐	☐	☐	☐
Schauenburger	☐	☐	☐	☐	☐
Star	☐	☐	☐	☐	☐
Summit	☐	☐	☐	☐	☐
Sweethart (= Sumtare)	☐	☐	☐	☐	☐
Techlovan	☐	☐	☐	☐	☐
Vanda	☐	☐	☐	☐	☐
Verarbeitung- und Schüttelkirschen	☐	☐	☐	☐	☐
Brennkirschen	☐	☐	☐	☐	☐
Andere	☐	☐	☐	☐	☐

5 Wie ist Ihre Anlage bezüglich Witterungsschutz und Insektenschutznetz konzipiert?

Bitte geben Sie Ihre Antwort sortenspezifisch an:

	Hagelschutz	Regendach	Vogelschutz	Insektennetz seitlich	Insektennetz oben und seitlich	Traufe eingenetzt	Vorhaupt eingenetzt
Bigarreau Burlat	☐	☐	☐	☐	☐	☐	☐
Carmen	☐	☐	☐	☐	☐	☐	☐
Christiana	☐	☐	☐	☐	☐	☐	☐
Gardel (Coralise®)	☐	☐	☐	☐	☐	☐	☐
Giorgia	☐	☐	☐	☐	☐	☐	☐
Grace Star	☐	☐	☐	☐	☐	☐	☐
Kordia	☐	☐	☐	☐	☐	☐	☐
Merchant	☐	☐	☐	☐	☐	☐	☐
Oktavia	☐	☐	☐	☐	☐	☐	☐
Regina	☐	☐	☐	☐	☐	☐	☐
Schauenburger	☐	☐	☐	☐	☐	☐	☐
Star	☐	☐	☐	☐	☐	☐	☐
Summit	☐	☐	☐	☐	☐	☐	☐
Sweethart (= Sumtare)	☐	☐	☐	☐	☐	☐	☐
Techlovan	☐	☐	☐	☐	☐	☐	☐
Vanda	☐	☐	☐	☐	☐	☐	☐
Verarbeitung- und Schüttelkirschen	☐	☐	☐	☐	☐	☐	☐
Brennkirschen	☐	☐	☐	☐	☐	☐	☐
Andere	☐	☐	☐	☐	☐	☐	☐

Wie gross ist die Maschenweite des Insektennetzes?

(Mehrfachnennungen sind möglich)

☐ 0.4 bis 0.8 mm (inkl. 0.8 x 0.8 mm)

- ☐ 0.8 bis 1.3 mm (inkl. 1.3 x 1.3 mm)
- ☐ 1.3 bis 1.7 mm (inkl. 1.7 x 1.7 mm)
- ☐ Bei gröberen Netzen Maschenweite angeben: ____

6 Wie gross schätzen Sie 2016 den Befall pro Sorte bei der Ernte (in Prozent)? (Pflichtfrage)

Haben Sie die Ernte abgebrochen?

	0 %	1 bis 10 %	Mehr als 10 %	Ernte abgebrochen
Bigarreau Burlat	☐	☐	☐	☐
Carmen	☐	☐	☐	☐
Christiana	☐	☐	☐	☐
Gardel (Coralise®)	☐	☐	☐	☐
Giorgia	☐	☐	☐	☐
Grace Star	☐	☐	☐	☐
Kordia	☐	☐	☐	☐
Merchant	☐	☐	☐	☐
Oktavia	☐	☐	☐	☐
Regina	☐	☐	☐	☐
Schauenburger	☐	☐	☐	☐
Star	☐	☐	☐	☐
Summit	☐	☐	☐	☐
Sweethart (= Sumtare)	☐	☐	☐	☐
Techlovan	☐	☐	☐	☐
Vanda	☐	☐	☐	☐
Verarbeitung- und Schüttelkirschen	☐	☐	☐	☐
Brennkirschen	☐	☐	☐	☐
Andere ____	☐	☐	☐	☐

7 Haben Sie die Kirschen nach der Ernte normal sortiert, oder haben Sie wegen der Kirschessigfliege (KEF) zusätzlichen Aufwand betreiben müssen?

	Ernte normal sortiert	Zusätzlicher Aufwand beim Sortieren wegen KEF
Bigarreau Burlat	☐	☐
Carmen	☐	☐
Christiana	☐	☐
Gardel (Coralise®)	☐	☐
Giorgia	☐	☐
Grace Star	☐	☐
Kordia	☐	☐
Merchant	☐	☐
Oktavia	☐	☐
Regina	☐	☐
Schauenburger	☐	☐
Star	☐	☐
Summit	☐	☐

Sweethart (= Sumtare)	☐	☐
Techlovan	☐	☐
Vanda	☐	☐
Verarbeitung- und Schüttelkirschen	☐	☐
Brennkirschen	☐	☐
Andere	☐	☐

8 Wie zeichnete sich der Befall der Kirschessigfliege aus?

(Mehrfachnennungen sind möglich)

☐ Nachgewiesene Eiablage
☐ Nachgewiesene Larve/Puppe
☐ Einstichlöcher
☐ Tropfende Früchte
☐ Essiggeruch
☐ Andere

9 Wurde die Kirschenqualität bei der Lieferung im Jahr 2016 wegen Kirschessigfliegen-Befall beanstandet?

	Ja	Nein
Bigarreau Burlat	☐	☐
Carmen	☐	☐
Christiana	☐	☐
Gardel (Coralise®)	☐	☐
Giorgia	☐	☐
Grace Star	☐	☐
Kordia	☐	☐
Merchant	☐	☐
Oktavia	☐	☐
Regina	☐	☐
Schauenburger	☐	☐
Star	☐	☐
Summit	☐	☐
Sweethart (= Sumtare)	☐	☐
Techlovan	☐	☐
Vanda	☐	☐
Verarbeitung- und Schüttelkirschen	☐	☐
Brennkirschen	☐	☐
Andere	☐	☐

10 Welche Strategien haben Sie gegen die Kirschessigfliege eingesetzt?

(Mehrfachnennungen sind möglich)

☐ Befallskontrolle
☐ Insektizideinsatz
☐ Hygienemassnahmen bei der Ernte
☐ Andere

11 Wie haben Sie die Befallskontrolle durchgeführt? (Pflichtfrage)

(Mehrfachnennungen sind möglich)

Visuelle Kontrolle

- ☐
- ☐ Salztest
- ☐ Seifen-/ Warmwasserkontrolle
- ☐ Andere

12 Insektizideinsatz: Welche Wirkstoffe haben Sie eingesetzt? (Pflichtfrage)

*oder andere Handelsprodukte mit gleichem Wirkstoff

(Mehrfachnennungen sind möglich)

- ☐ Thiacloprid (z.B Alanto*)
- ☐ Spinosad (z.B Audienz*)
- ☐ Acetamiprid (z.B Gazelle SG*)
- ☐ Pyrethrine (z.B Parexan N* oder Pyrethrum FS*)
- ☐ Andere

13 Welche Hygienemassnahmen haben Sie getroffen? (Pflichtfrage)

(Mehrfachnennungen sind möglich)

- ☐ Unterwuchs tief gehalten (gemäht/gemulcht)
- ☐ Vorzeitiger Erntebeginn
- ☐ Sauberes Abernten jeder Sorte (keine hängengelassene Früchte)
- ☐ Ernteabgang aus der Anlage entfernt
- ☐ Ernteabgang gemulcht
- ☐ Nacherntebehandlung
- ☐ Andere

14 Was für eine Nacherntebehandlung haben Sie durchgeführt?

15 Wie gross ist Ihre Zufriedenheit mit den von Ihnen ausgewählten Strategien pro Sorte?

Zufriedenheit (5=sehr zufrieden, 1=sehr unzufrieden)

	5 (sehr zufrieden)	4	3	2	1 (sehr unzufrieden)
Bigarreau Burlat	○	○	○	○	○
Carmen	○	○	○	○	○
Christiana	○	○	○	○	○
Gardel (Coralise®)	○	○	○	○	○
Giorgia	○	○	○	○	○
Grace Star	○	○	○	○	○
Kordia	○	○	○	○	○
Merchant	○	○	○	○	○
Oktavia	○	○	○	○	○
Regina	○	○	○	○	○
Schauenburger	○	○	○	○	○
Star	○	○	○	○	○
Summit	○	○	○	○	○
Sweethart (= Sumtare)	○	○	○	○	○
Techlovan	○	○	○	○	○
Vanda	○	○	○	○	○

Verarbeitung- und Schüttelkirschen	○	○	○	○	○
Brennkirschen	○	○	○	○	○
Andere ______	○	○	○	○	○

16 Wenn Sie nicht zufrieden sind, was werden Sie in Zukunft anders machen?

17 Wo holen Sie sich Ihr Fachwissen zur Kirschessigfliege?

(Mehrfachnennungen sind möglich)

- ☐ Merkblätter und Informationen von Agroscope
- ☐ Merkblätter und Informationen der kantonalen Fachstellen
- ☐ Breitenhoftagung
- ☐ Kantonale Obstbautagungen
- ☐ Andere Tagungen
- ☐ Fachartikel/Fachzeitschriften
- ☐ Pflanzenschutzmitteilungen Obst- und Rebbau
- ☐ AGRIDEA
- ☐ Im Internet googlen
- ☐ Andere Produzenten
- ☐ Andere ______

18 Gibt es an einer Distanz von weniger als 100 m von Ihren Anlagen folgende Elemente? Wie beeinflussen, Ihrer Meinung nach folgende Elemente, den Kirschessigfliegen-Befall auf Ihrem Betrieb?

	**Elment vorhanden**	**kein Einfluss**	**mehr Befall**	**weniger Befall**	**weiss ich nicht**
Waldrand	☐	☐	☐	☐	☐
Hecken	☐	☐	☐	☐	☐
Gepflegte Hochstämme	☐	☐	☐	☐	☐
Ungepflegte Hochstämme	☐	☐	☐	☐	☐
Gewässer in der Nähe	☐	☐	☐	☐	☐
Obst- oder Beerenproduktion in der Nähe	☐	☐	☐	☐	☐
Rebberge in der Nähe	☐	☐	☐	☐	☐
Ungentutze Habitate	☐	☐	☐	☐	☐
Komposthaufen	☐	☐	☐	☐	☐
Andere ______	☐	☐	☐	☐	☐

19 Haben Sie besondere Festellungen über die Kirschessigfliege?

20 Fragen zu Ihren Betrieb

In welchem Kanton befindet sich Ihr Betrieb?

Bitte auswählen ▾

Welchen Schwerpunkt hat Ihr Betrieb?

- ○ Kirschenproduktion
- ○ Steinobstproduktion (Kirschen und Zwetschgen)
- ○ Kernobstproduktion
- ○ Kern- und Steinobstproduktion
- ○ Mischbetrieb Obstbau und Viehhaltung
- ○ Andere ______

Nach welcher Produktionsform bewirtschaften Sie?

- ○ ÖLN
- ○ Bio
- ○ Konventionell

Wie viele Standardarbeitskräfte (SAK) hat Ihren Betrieb? ______

Wie gross ist Ihre gesamte Betriebsfläche in Aren? ______

Welcher Anteil der Betriebsfläche pachten Sie?

- ○ 0-25%
- ○ 26-50%
- ○ 51-75%
- ○ 76-100%

Wie gross ist Ihre gesamte Kirschenfläche in Aren? ______

Wie vermarkten Sie Ihre Tafelkirschen (in %)?

Verkauf ab Hof	
Verkauf an Handel	
Verkauf direkt an Grossverteiler	
Verkauf an kleinere Fachgeschäfte	
Andere	

Haben Sie eine Hagelversicherung?

(Mehrfachnennungen sind möglich)

- ☐ Nein, ich habe keine Hagelversicherung
- ☐ Ja, ich habe die Hagelversicherung 'Fix' (Schweizer Hagel)
- ☐ Ja, ich habe die Hagelversicherung 'Standard' (Schweizer Hagel)
- ☐ Ja, ich habe die Hagelversicherung 'Erhöht' (Schweizer Hagel)

Welche der folgenden Risikomanagementstrategien setzen Sie auf Ihrem Betrieb ein?

(Mehrfachnennungen sind möglich)

- ☐ Landwirtschaftsnahe Diversifizierung (z.B. Agrotourismus)
- ☐ Tätigkeiten ausserhalb des Betriebes (z.B Beratung)
- ☐ Bildung von finanziellen Reserven (Sparen für schlechte Zeiten)
- ☐ Härter Arbeiten / private Ausgaben kürzen
- ☐ Investitionen ausserhalb des Betriebs (z.B andere Unternehmungen, Immobilien)
- ☐ Andere Strategien ______

21 Persönliche Fragen

Ihr Geschlecht

- ◯ Weiblich
- ◯ Männlich

Ihr Lebensalter

- ◯ bis 30 Jahre alt
- ◯ 31-40 Jahre alt
- ◯ 41- 50 Jahre alt
- ◯ über 50 Jahre alt

22 **Bitte geben Sie in der folgenden Tabelle an inwiefern Sie den Aussagen zustimmen (von 1 = „stimme voll zu“ bis 5 = „lehne ab“).**

	1 (stimme voll zu)	2	3	4	5 (lehne ab)
Ich bin bereit, in Bezug auf die **Produktion** mehr Risiken einzugehen als andere Landwirte.	☐	☐	☐	☐	☐
Ich bin bereit, in Bezug auf **Markt- und Preise** mehr Risiken einzugehen als andere Landwirte.	☐	☐	☐	☐	☐
Ich bin bereit, in Bezug auf **Fremdkapitalaufnahme** mehr Risiken einzugehen als andere Landwirte.	☐	☐	☐	☐	☐
Ich bin bereit, in Bezug auf **Landwirtschaft generell** mehr Risiken einzugehen als an-dere Landwirte.	☐	☐	☐	☐	☐

23 **Ist auf Ihrem Betrieb die Hofnachfolge gesichert?**

- ◯ Ja
- ◯ Eher schon
- ◯ Eher nicht
- ◯ Nein
- ◯ Nein, es steht keine Hofübernahme in den nächsten 15 Jahren an
- ◯ Nein, auslaufender Betrieb
- ◯ Andere [ ]

24 **Haushalteinkommen**

Wie viel des gesamten Haushalteinkommens beziehen Sie aus der landwirtschaftlichen Tätigkeit?

- ◯ weniger als 10%
- ◯ 10% - 30%
- ◯ 31% - 50%
- ◯ 51% - 70%
- ◯ mehr als 70%

Wie viel trägt die Tafelkirschenproduktion zu Ihren landwirtschaftlichen Einkommen bei?

- ◯ 0-25%
- ◯ 26-50%
- ◯ 51-75%
- ◯ 76-100%

25 **Um Ihre Risikobereitschaft und die Massnahmen die Sie auf Ihren Betrieb gegen die Kirschessigfliege einsetzten besser einzuordnen bitten wir Sie nun 7 Fragen zu Ihrem Umgang mit Zahlen und Wahrscheinlichkeiten zu beantworten:**

Wie schätzen Sie sich im Bruchrechnen ein?

- ◯ 1 (überhaupt nicht gut)
- ◯ 2
- ◯ 3
- ◯ 4
- ◯ 5
- ◯ 6 (sehr gut)

Wie schätzen Sie Ihre Fähigkeiten im Umgang mit Prozentwerten ein?

- ○ 1 (überhaupt nicht gut)
- ○ 2
- ○ 3
- ○ 4
- ○ 5
- ○ 6 (sehr gut)

Wie gut sind Sie darin einzuschätzen, wie viel eine 25%-ge Preisreduzierung bedeutet?

- ○ 1 (überhaupt nicht gut)
- ○ 2
- ○ 3
- ○ 4
- ○ 5
- ○ 6 (sehr gut)

Wenn Sie eine Tageszeitung lesen, wie nützlich finden Sie Tabellen und Diagramme als Teil eines Artikels?

- ○ 1 (überhaupt nicht nützlich)
- ○ 2
- ○ 3
- ○ 4
- ○ 5
- ○ 6 (sehr nützlich)

Wenn Ihnen jemand etwas über die Wahrscheinlichkeit erzählt, dass ein bestimmtes Ereignis eintreffen wird, bevorzugen Sie es dann, wenn dazu Worte benutzt werden („passiert selten“) oder wenn Zahlenwerte benutzt werden („es gibt eine 1%ige Wahrscheinlichkeit“)?

- ○ 1 (bevorzuge immer Prozentwerte)
- ○ 2
- ○ 3
- ○ 4
- ○ 5
- ○ 6 (bevorzuge immer Worte)

Wenn Sie einen Wetterbericht hören, bevorzugen Sie es dann, wenn die Vorhersagen in Prozentwerten ausgedrückt werden (z.B. „es gibt heute eine 20%ige Regenwahrscheinlichkeit“) oder in Worten(z.B. „heute ist die Regenwahrscheinlichkeit gering“)?

- ○ 1 (bevorzuge immer Worte)
- ○ 2
- ○ 3
- ○ 4
- ○ 5
- ○ 6 (bevorzuge immer Prozentwerte)

Wie oft finden Sie Informationen, die in Zahlen ausgedrückt sind, nützlich?

- ○ 1 (nie)
- ○ 2
- ○ 3
- ○ 4
- ○ 5
- ○ 6 (sehr oft)

26 **Um Ihren Befall mit Wetterdaten Ihrer Region zu verknüpfen bitten wir Sie hier Ihre Adresse zu hinterlassen (frewillig):**

Strasse: ____________________

PLZ: [ ]

27 Schlussfragen

Möchten Sie an der Verlosung von vier SBB-Gutscheine in Wert von 50 CHF teilnehmen? Wenn ja, geben Sie unten Ihre E-Mail Adresse an:

- ○ Ja
- ○ Nein

Möchten Sie die Ergebnisse der Umfrage erhalten? Wenn ja, geben Sie unten Ihre E-Mail Adresse an:

- ○ Ja
- ○ Nein

Ihre E-Mail Adresse: [ ]

Vielen Dank für Ihre Teilnahme. Sie können den Internet-Browser jetzt schließen.
